# Full-Thickness Craniodural Metastasis with Leptomeningeal Infiltration of Salivary Origin: A Radiological Lesson and a Technical Remark

**DOI:** 10.3390/tomography8050181

**Published:** 2022-08-27

**Authors:** Alessandro Pesce, Daniele Armocida, Francesco Fiorentino, Silvia Ciarlo, Biagia La Pira, Maurizio Salvati, Alessandro Frati, Angelo Pompucci, Mauro Palmieri

**Affiliations:** 1 Neurosurgery Division, Santa Maria Goretti Hospital, 04100 Latina, Italy; 2 Neurosurgery Division, A.O.U. “Policlinico Umberto I”, Human Neuroscience Department, “Sapienza” University, Viale del Policlinico 155, 00161 Rome, Italy; 3 Pathology Division, Santa Maria Goretti Hospital, 04100 Latina, Italy; 4 Neurosurgery Department, A.O.U. ‘Mater Domini’, Università degli Studi ‘Magna Greacia’ di Catanzaro, 88100 Catanzaro, Italy; 5 Neurosurgery Department, Azienda Ospedaliera Fabrizio Spaziani, 03100 Frosinone, Italy; 6 Department of Neurosurgery, Policlinico “Tor Vergata”, University of Rome ‘‘Tor Vergata”, 00133 Rome, Italy; 7 IRCCS—“Neuromed”, Via Atinense 18, 86077 Pozzilli, Italy

**Keywords:** calvarial metastasis, salivary gland tumors, craniotomy, dural metastasis

## Abstract

Calvarial metastases are a relatively rare entity, with an overall incidence of 3–4%. Among these cases, metastases arising from salivary gland cancers are even rarer; in fact, large studies regarding salivary gland tumors showed that brain metastases are observed in 0.8% of the cases. Generally, bone metastases have been described in proximity to primary tumors, while bloodstream-disseminated lesions are often located inside the brain parenchyma. During every surgical step, traction on lower-lying infiltrated tissues must be avoided in order to successfully remove the lesion. This case report presents the first ever case of a 67-year-old woman affected by submandibular gland undifferentiated adenocarcinoma metastasis with a full-thickness involvement of the calvarium, pachy- and leptomeninges.

## 1. Introduction

The overall incidence of calvarial metastases is as high as 15–25% [1], the dural metastases being relatively rarer with an overall incidence of 3–4% [2]. Globally, their incidence is progressively increasing because of the increased life expectancy of patients suffering from metastatic malignant diseases. Metastases spread to the calvarium via three routes: via Batson’s venous plexus, hematogenous spread or direct extension through the cranial foramina [3]. In the case of calvarial and dural invasion, either the bony metastasis involving the cranium infiltrates the dural plane (57% of cases) [4] or a purely dural metastasis promotes the infiltration of the calvarial surface. Clinical manifestations of such conditions are simple cranial “lumps”, fixed on the over- and underlying layers, seizures, and neurological deficits [4,5]. The radiological gold standard for the anatomical understanding of the lesion and its relationship with the dural and pial surfaces is reached with the combination of MRI [5] and CT scans. Although the skull involvement via direct extension of infiltrating malignancies of salivary origin is more common [6,7], and several treatment modalities were advocated for the management of such conditions [1,7], to the best of our knowledge this is the first ever reported case of a submandibular gland undifferentiated adenocarcinoma metastasis with a full-thickness involvement of the calvarium, pachy- and leptomeninges. Apart from the pathological exceptionality of this case, the report presents several specific features of critical importance in the surgery of the calvarial-dural metastases which are, in our opinion, worth reporting and discussing. 

## 2. Case Description

**Figure 1:**
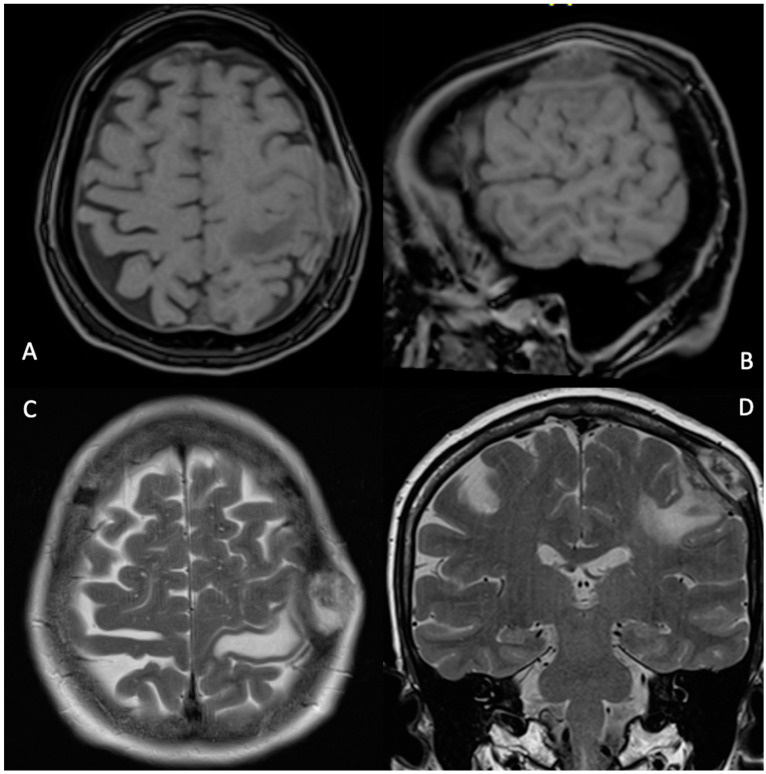
MRI (**A**) axial and (**B**) sagittal T1-weighted scans showing intra-calverian metastasis; (**C**) axial and (**D**) sagittal T2-weighted scans showing the lesion and the related edema in motor area.

**Figure 2:**
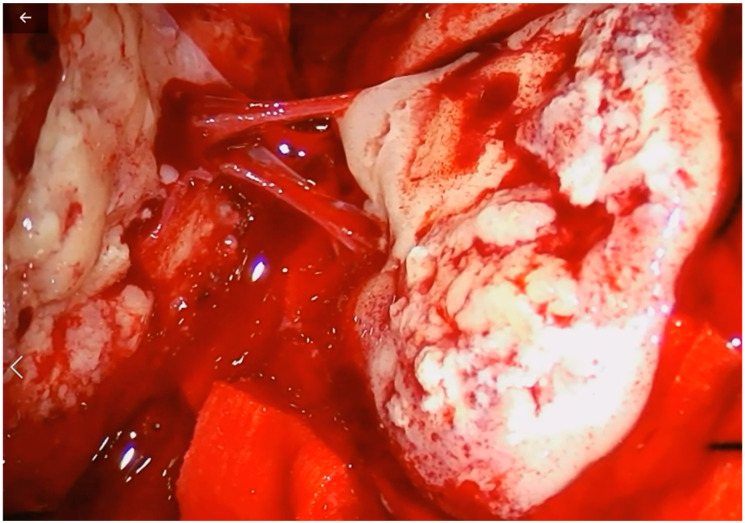
Cortical vein involved in focal thrombosis due to surgical manipulation.

**Figure 3:**
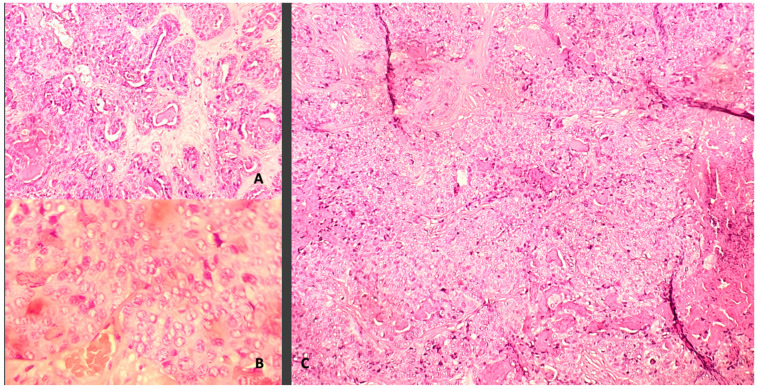
Adenocarcinoma with glandular differentiation (**A**) and with solid and necrotic areas (**B**). At higher magnification, (**C**) note pleomorphic nuclei, eosinophilic nucleoli and mitotic figures.

**Figure 4:**
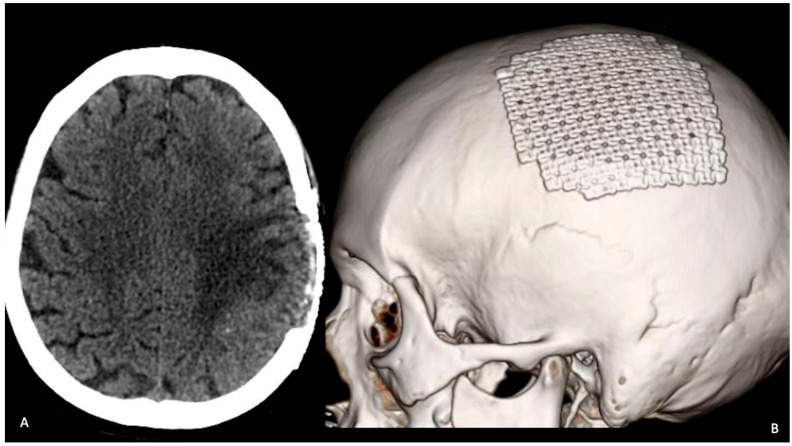
(**A**) CT axial scan performed 30 days after surgery showing good radiological outcome. (**B**) 3D detail of the mesh used for skull reconstruction.

A 67-year-old woman came to our emergency department complaining of a sudden onset right hand numbness and slurred speech. She had a previous history of left submandibular gland excision and monolateral ispilateral left-radical neck dissection for an undifferentiated salivary adenocarcinoma (pT2, pN2, CK 5,6,7 and 19+, p53+, TTF1-, NapsinA-, ER-, c-Erb2-, S100+/−, Ki67 15–20%). Additionally, she underwent a schedule of adjuvant treatments composed of local radiation therapy, focused on the small metastatic foci identified with the total-body CT scan: cervical nodes, oral cavity, subpleural, surrenal gland and acetabular and a conventional chemotherapy (CDDP). This was followed, later by a biological therapy regime (Tas-120, 4 mg), with a clinical result of a cumulative 4 years of progression-free survival. The general history demonstrated an anaphylactic allergy to several drugs including iodine and gadolinium contrast media. The physical examination disclosed a slight right-hand weakness and numbness, with impairmea nt of the fine movements of the hands. The cranial surface examination revealed a lump of hard consistency, fixed on the deep and superficial layers. The preoperative brain CT and MRI scan could not be performed with contrast mediums. It revealed the presence of an osteolytic rounded lesion with irregular margins, involving in full thickness from the subcutaneous tissues to the dura, while the involvement of the leptomeningeal layer was not surely outlined. The diameters were 27 × 25 × 15 mm. T2w sequence and FLAIR disclosed the presence of a rolandic finger-shaped edema, together with an hyperintense dishomogeneous pattern on the lesion (Figure 1). The arterial and venous cortical structures were of course impossible to visualize. The patient was operated on with general anesthesia protocol, without muscle relaxants, for the need of the intraoperative neuromonitoring. After a c-shaped skin incision, centered over the lump, the lesion was identified and divided from its subcutaneous/galeal extension. The craniotomy included approximately 3–3.5 cm of bony margin, which was apparently free of the disease; the craniotomy itself was subsequently concluded with four further “cross-shaped” cuts to avoid any traction on the cortical surface during the elevation of the flap. The dura was incised in a circular fashion. An obvious and extensive violation of the leptomeningeal and cortical layers was found while elevating the dura flap: a vein coursed inside the tumor was carefully dissected, and the distalmost segment entering the lesion was coagulated and divided. After a complete resection a titanium mesh completed the reconstruction. After surgery, the patient presented a transient worsening of the speech function with an obvious motor aphasia which lasted overall 3 days. Such an occurrence was probably related to a manipulation-related focal thrombosis of the cortical vein (Figure 2). After the fourth postoperative day, the speech function started significantly improving and on 8th postoperative day the patient was discharged in good general conditions, without neurological impairment. The histological exam confirmed the diagnosis (Figure 3). A CT scan at the 30th postoperative day disclosed no bleeding and a good general condition of the surgical field (Figure 4). Neurological examinations at 30 and 120 days were stable and normal. After surgery, which comprised a radical resection of the lesion, the foregoing adjuvant treatment was continued. 

## 3. Discussion

Salivary gland tumors are rare and divided into several histopathological groups, generally affecting the parotid gland in near the 70% of the cases [8], while, consequently, the submandibular glands are involved in fewer cases. Moreover, malignant salivary glands tumors are even less common than the benign ones, accounting for 20% of the cases [8,9]. Seldom, papers in the whole of the English literature reported rare cases of intracranial metastasis deriving from salivary gland tumors [9,10,11,12,13,14,15,16,17,18,19,20,21,22,23,24,25,26,27,28]. More frequent localization of metastases deriving from salivary glands tumors are lung, bone, liver and skin [29]. In fact, large studies regarding salivary gland tumors showed that brain metastases are observed in the 0.8% of the cases [9], in both autopsies and surgical series. Among these, in only one other paper a case of cranio-calvarialmetastasis [9] deriving from a parotid gland tumor was reported; in the other reports, all the secondary intracranial localization regarded intraparenchymal lesions. Therefore, this is the first case of a cranio-calvarialmetastasis deriving from an adenocarcinoma of submandibular salivary glands. As stated before, the localization in bony structures is rare, and for this kind of lesion is more often described when the metastasis occurs in the primary tumor with a contiguous mechanism of dissemination [9]. In this case, the metastasis is not located near the salivary glands or in the neck area; therefore, it can be supposed that the dissemination of the primary tumor occurred through bloodstream dissemination, even though the reported cases of bloodstream-disseminated intracranial metastasis from salivary glands cancer are generally located intraparenchymally [9,14,15]. 

This case offers several points of reflection. First of all, the patient suffered from severe allergy to multiple drugs; in this condition, since she was allergic also to gadolinium contrast medium, we did not have an unquestionable imaging disclosing the infiltration of the leptomeninges and cortex, although the preoperative MRI was highly suspicious (Figure 1). In such fortunately rare cases, we find useful to plan a wider, possibly C-shaped skin incision. This is because, as in the present case, there could be the need to broaden the craniotomic flap to encompass all the macroscopically infiltrated area while possibly leaving an apparently safe bony margin, and to reduce the risk of incomplete resection [9] which, whenever feasible is always advisable. In case of suspected full-thickness infiltration, a further fragmentation of the flap is advisable to minimize or avoid any traction on the underlying levels. Moreover, the important message is that in cases of full-thickness infiltration, the small venous collectors are at high risk of being critical cortical branches, even when encased by pathologic tissue and directly entering the lesion. A careful dissection should be carried on in order to try to preserve such veins.

## 4. Conclusions

Undifferentiated carcinomas of salivary origins can manifest as solitary skull metastasis: either, with an obvious potential to infiltrate the surrounding tissue deep into the cortex and outwards into the subcutaneous tissue; or with an insidious potential to encase and hemodynamically hijack small venous and possibly arterial collectors. Careful dissection may avoid the sacrifice of such important structures.
